# Clinicopathological Characteristics of Thyroid Cancer in a Saudi Academic Hospital

**DOI:** 10.7759/cureus.8044

**Published:** 2020-05-10

**Authors:** Shaza Samargandy, Rahaf Qari, Arwa Aljadani, Doaa Assaqaf, Abdulaziz Etaiwi, Doaa Alghamdi, Hani Marzouki, Amani Alhozali, Mazin Merdad, Marwan Al-Hajeili

**Affiliations:** 1 Endocrinology, King Abdulaziz University Hospital, Jeddah, SAU; 2 Internal Medicine, King Abdulaziz University Hospital, Jeddah, SAU; 3 Head and Neck Pathology, King Abdulaziz University Hospital, Jeddah, SAU; 4 Head and Neck Surgery, King Abdulaziz University Hospital, Jeddah, SAU; 5 Oncology, King Abdulaziz University Hospital, Jeddah, SAU

**Keywords:** thyroid cancer, malignancy, epidemiology, single centre study

## Abstract

Background

The global incidence of thyroid cancer (TC) has increased significantly over the past decades. In Saudi Arabia, it is the third most common cancer among adults. This study aims to review the clinical and histopathological characteristics of TC in Saudi Arabia and analyze the size trend over the years.

Methods

We conducted a retrospective chart review of all differentiated and poorly differentiated TC patients following up at a tertiary care center in the Western region of Saudi Arabia. All patients 11 years and older, diagnosed between 2004 - 2018, and with sufficient histopathological data were included.

Follicular and poorly differentiated TC were categorized and tumor stage was allocated. We performed descriptive and size trend analysis.

Results

We had a total of 285 patients who qualified for inclusion. The mean age at diagnosis was 40.6 years, and 81.05% of patients were females. Papillary TC comprised 88.07% of these neoplasms, and most patients (89.12%) were at Stage I. Only papillary TC showed a gender difference in the age of the diagnosis. In men, the mean age at diagnosis of papillary TC was 46.98 ± 15.4 years, while in female patients, it was 39.02 ± 12.8 years (p-value = 0.0001).

We did not find a trend toward smaller tumours in the more recent years in comparison to the early years (r = -0.083, p-value = 0.168).

Conclusions

TC is diagnosed at a younger age and larger sizes in Saudi Arabia in comparison to other countries. A gender difference was only noted with papillary TC in regard to the age of diagnosis. There was no trend toward smaller sizes of TC over the years.

## Introduction

The global incidence of thyroid cancer (TC) has increased significantly over the past decades. It was postulated that this is mainly due to overdiagnosis attributed to advances in medical care, diagnostic imaging, and fine-needle aspiration cytology studies [1-6]. However, recent epidemiological studies suggest an actual rise in the incidence of TC. In a 39-year-retrospective study using the United States (US) Surveillance, Epidemiology, and End Results-9 (SEER-9) database, the incidence of TC has increased by 3% every year [7].

In Saudi Arabia, according to the most recent Saudi Cancer Registry Report, TC is the second most common malignancy among females and the ninth among males [8]. Comparing the incidence of TC in Saudi Arabia from 1990 until 2016, there was a 26-fold surge in the incidence of this disease, and the increase of the Saudi population did not solely explain this [9]. Saudi literature harbours a limited number of publications in the field of TC with a clear gap in the knowledge of poorly differentiated TC and the epidemiology of this disease, especially in the Western part of Saudi Arabia [8].

With the relatively recent updates in the American Joint Committee on Cancer (AJCC) staging classification and the World Health Organization (WHO) pathology classification of endocrine tumors, it has become essential to analyse TC epidemiology in Saudi Arabia in the view of these new changes [10-11].

Herein, we review the clinical and histopathological characteristics of differentiated and poorly differentiated TC over 14 years in an oncology referral centre in Saudi Arabia. We have also analysed the size trend over the years to investigate whether we would have a trend toward smaller sizes corresponding to the surge of medical imaging.

## Materials and methods

We conducted a single-centre, retrospective electronic chart review of all differentiated and poorly differentiated TC patients over a 14 year period. We included patients aged 11 years and older who were diagnosed between 2004 and 2018 with sufficient histological data and complete tumour-node-metastasis (TNM) staging information. For each patient, we collected the following variables: gender, age at diagnosis, tumour stage at the time of diagnosis, and the tumour’s histological details. The type of surgery performed and whether the patient received radioactive iodine therapy were also documented.

The histological patterns included in our study were papillary, follicular, Hürthle cell, and poorly differentiated TC, in addition to noninvasive follicular thyroid neoplasms with papillary-like nuclear features (NIFTP) cases. The nomenclature of NIFTP was adapted in our centre in 2017. We did not perform a retrospective review of encapsulated cases prior to 2017, and NIFTP was not included in the tumour staging as per the most recent American Thyroid Association statement and recommendations regarding NIFTP [12].

Tumour stage was determined based on the eighth edition of the AJCC/TNM Staging System for Differentiated and Anaplastic Thyroid Cancer [13]. Regarding histological subtypes, the most recent WHO Classification Pathology of Endocrine Tumours - 2017 Update was applied in our institute in January 2018, and all cases afterward were categorized accordingly [11]. Each follicular, Hürthle cell and poorly differentiated TC cases before 2018 were reviewed and reported based on the 2017 WHO classification [11]. All papillary TCs measuring 1 cm or less in size were labeled as microcarcinoma variants as per the recent WHO classification. No retrospective review was done before 2018 for the rest of the papillary TC variants. We excluded patients with medullary or anaplastic thyroid cancers and primary thyroid lymphomas. Patients with missing clinical or histopathological data were also excluded. 

In the present study, statistical analysis using the IBM Statistical Package for Social Sciences (SPSS), version 20.0 (IBM SPSS Statistics, Armonk, NY) was applied to evaluate and test the hypothesis. The Shapiro-Wilk test was used to test the normality of age in years across groups of pathology and gender. Pearson’s correlation was used to measure the strength and direction of association that existed between the year of diagnosis and tumour size. Independent sample T-test analysis was applied to determine whether there was statistical evidence that the mean age difference between genders was significant.

## Results

A total of 285 patients were ultimately enrolled after the exclusion criteria were applied. Of those, 231 (81.05%) were females and 54 (18.95%) were males. The mean age at diagnoses was 40.7 years. Papillary TC comprised the majority of these malignancies, with a total of 251 patients (88.07%). Baseline characteristics of the patients, stage at diagnosis, and management are summarized in Table 1.

**Table 1:** Patient Characteristics at Baseline *Tumour size data was missing on 14 patients so the mean was calculated on the data available for 271 patients. NIFTP: noninvasive follicular thyroid neoplasm with papillary-like nuclear features; RAI: radioactive iodine; SD: standard deviation

Variable		Total	Percent (%)
		n = 285	Mean ± SD
Sex	Male	54	18.95%
	Female	231	81.05%
Mean age at diagnosis (years) ± SD		285	40.66 ± 14.18
Pathology	Papillary	251	88.1%
	Follicular	18	6.3%
	Poorly differentiated	8	2.8%
	Hürthle cell	6	2.1%
	NIFTP	2	0.7%
Mean tumor size (cm) ± SD*		271	2.33 ± 1.84
Stage at diagnosis	Not applicable (NIFTP)	2	0.70%
	I	254	89.12%
	II	20	7.02%
	III	1	0.35%
	IVb	8	2.81%
Type of surgery	Lobectomy/hemithyroidectomy	25	8.77%
	Total thyroidectomy/near-total thyroidectomy	260	91.23%
Lymph node dissection	Done	78	27.66%
RAI	None	100	35.09%
	Received	185	64.91%

For all histological types, female patients predominated the number of patients. Female patients were 81.3% of all patients with papillary TC, 83.3% of all follicular TC, 75% of all poorly differentiated TC, 66.7% of all Hürthle cell TC, and all of the NIFTP cases.

**Figure 1:**
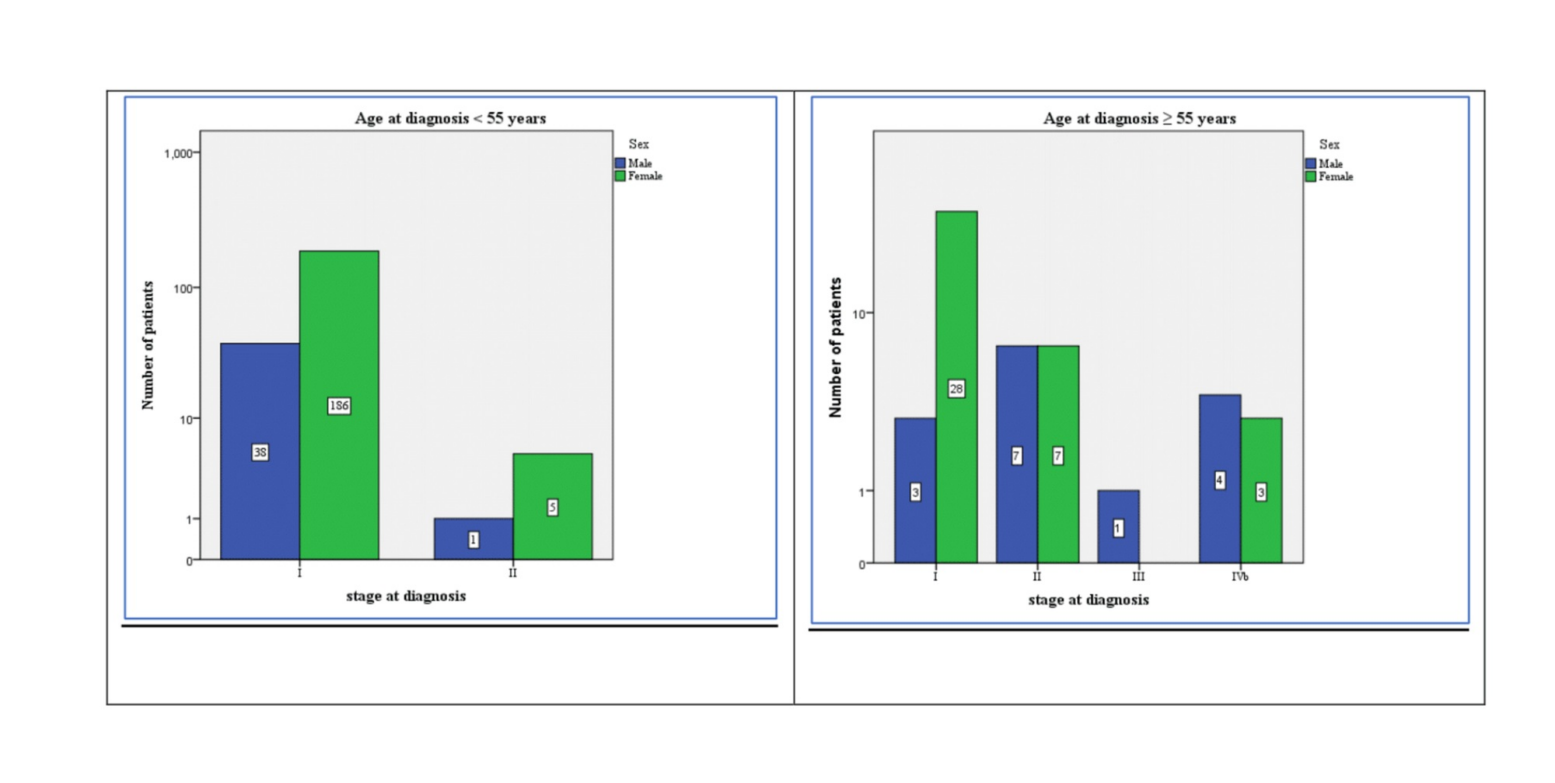
Demographics and cancer stages of the patients* * noninvasive follicular thyroid neoplasm with papillary-like nuclear features (NIFTP) patients were not included in the staging system

The majority of patients (81.4%) were young (below the age of 55 years), and most of those patients (97.4%) were diagnosed with Stage I cancers. Older patients who were 55 years or more (58.5%) were also less commonly diagnosed at Stage I in comparison to younger patients. In addition, metastatic disease at diagnosis was more common in older patients (13.2% vs. 2.6%) (Figure 1).

**Figure 2:**
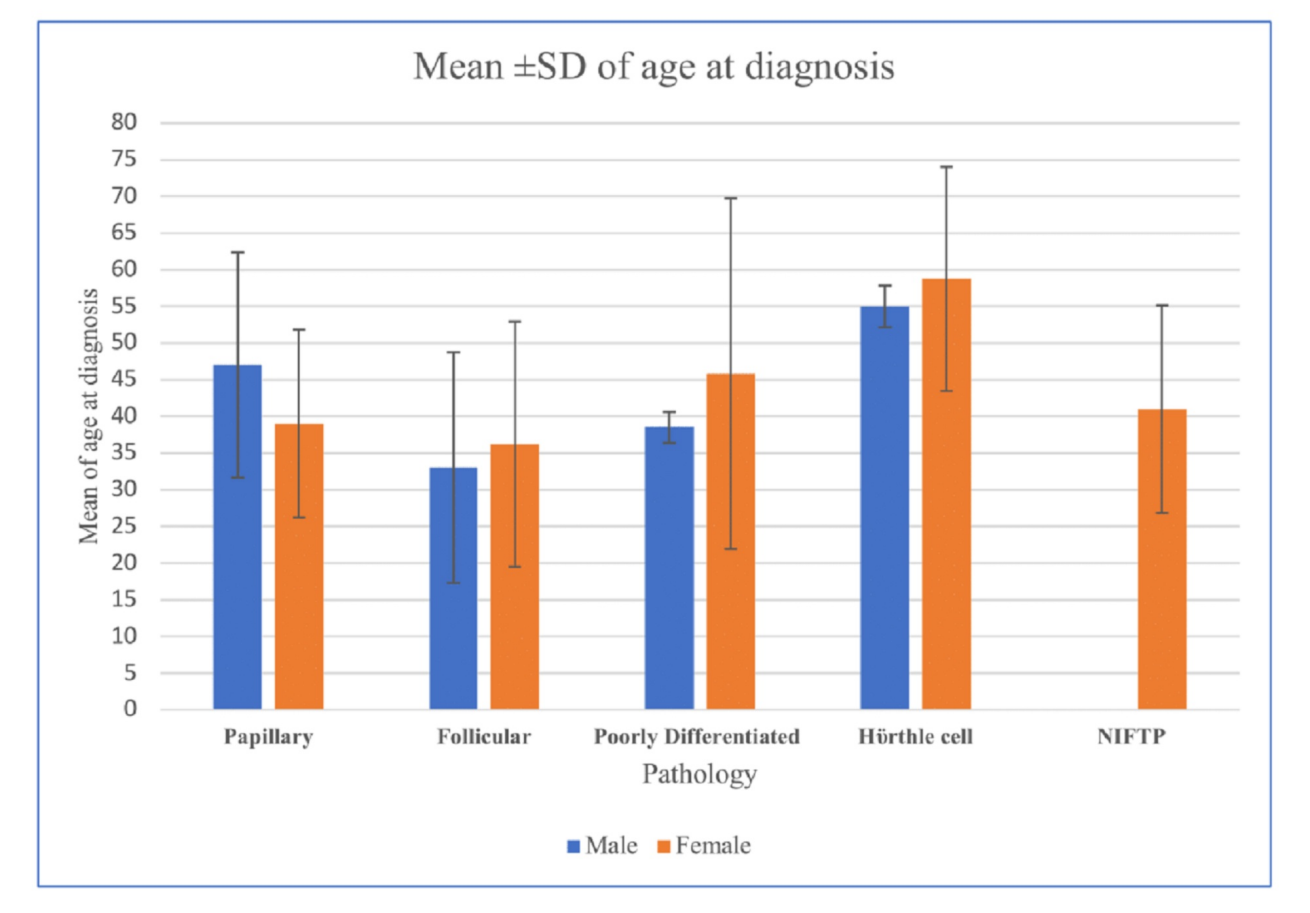
Thyroid cancer incidence trends by sex, age, and pathology p-value for papillary TC = 0.0001; p-value for follicular TC = 0.76; p-value for poorly differentiated TC = 0.69; p-value for Hürthle cell TC = 0.76 NIFTP: noninvasive follicular thyroid neoplasm with papillary-like nuclear features (not applicable); SD: standard deviation; TC: thyroid cancer

Male patients diagnosed with papillary TC were older (mean age: 46.98 ± 15.4) in comparison to female papillary TC patients (mean age: 39.02 ± 12.8) with a mean difference of 7.96 years (95% CI 3.71 - 12.20). For the rest of the histopathological types, there was no significant age difference between male and female patients. Patients with Hürthle cell carcinoma were generally older than the rest of the histological types (Figure 2).

From the 251 papillary TC patients included in the cohort, 183 had a documented histological variant. Papillary microcarcinoma comprised the majority of them (38.3%). For follicular TC, the most prevalent subtype was encapsulated angioinvasive subtype which constituted 44.4% of all follicular TC cases. The detailed histopathological features are further reviewed in Tables 2-5.

**Table 2:** Papillary Thyroid Cancer Histological Variants

Valid percent (%)	Frequency	Variant
30.6	56	Classical
24.6	45	Follicular
3.3	6	Tall cell variant
0.5	1	Solid
0.5	1	Columnar
38.3	70	Papillary microcarcinoma
1.6	3	Oncocytic
0.5	1	Warthin-like
100	183	Total

**Table 3:** Follicular Thyroid Carcinoma Histological Subtypes

Subtype	Frequency	Valid percent (%)
Minimally invasive	5	27.8
Encapsulated angio-invasive	8	44.4
Widely invasive	5	27.8
Total	18	100

**Table 4:** Thyroid Cancer Histological Characteristic

Histological characteristic	Number (valid percent)
Vascular invasion	49 (19.3%)
Extrathyroidal extension	42 (15.6%)
Multifocality	117 (42.7%)
Lymph node metastasis	71 (24.9%)
Largest tumor focus > 4 cm	43 (15.9%)
Positive margins	51 (21.5%)
Capsule invasion	69 (33.5%)

**Table 5:** Extent of Extrathyroidal Extension (ETE) ^1^ Detected only on histological examination ^2^ Detected intraoperatively and on histological examination

Degree	Frequency	Valid percent (%)
None (intrathyroidal)	227	84.4
Minimal ETE^1^	33	12.3
Gross ETE^2^	9	3.3
Total	269	100

**Figure 3:**
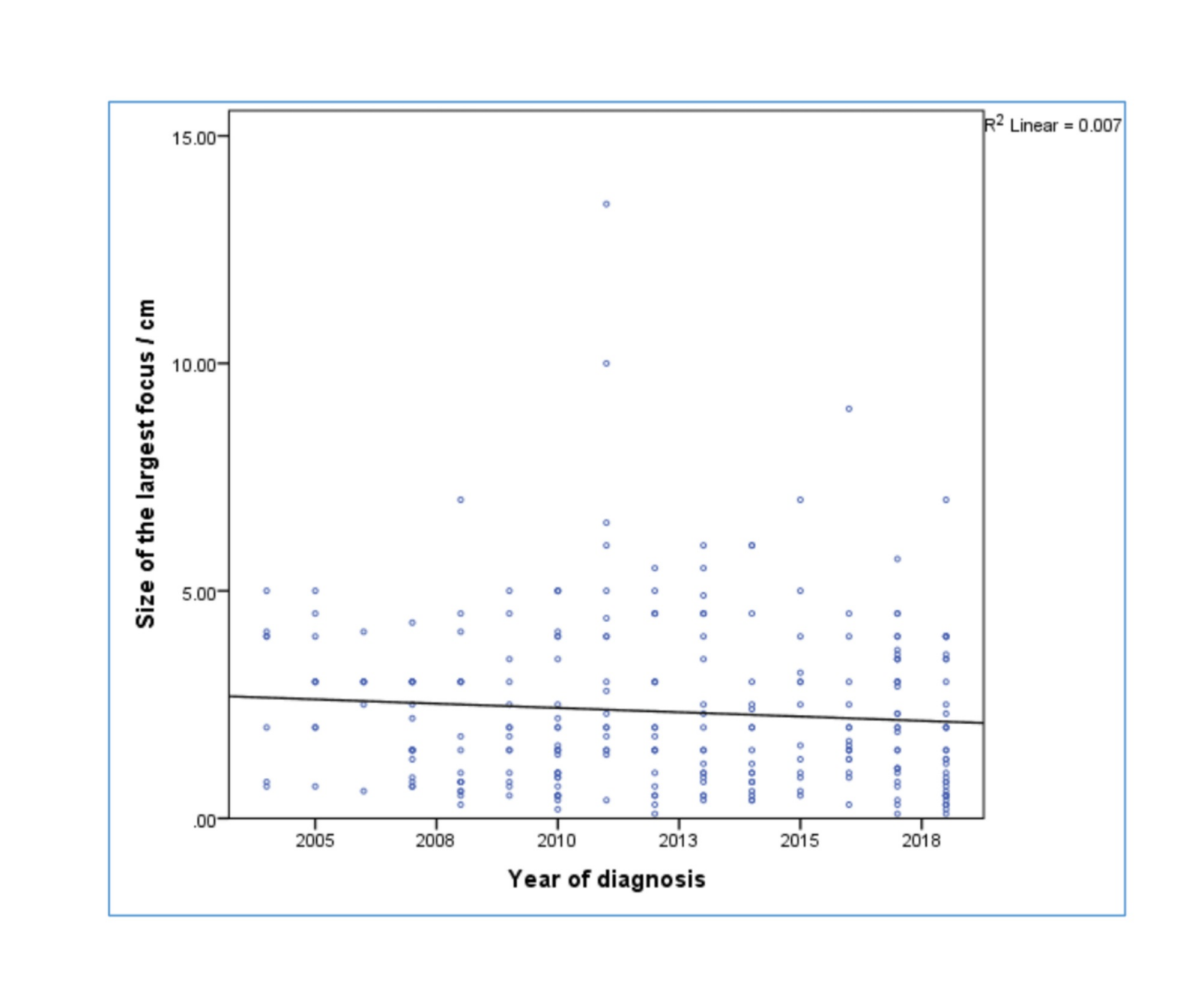
Correlation between the year of diagnosis and the tumor size

The mean tumour size was 2.7 cm, and the largest size recorded in the registry was 13 cm. Contrary to the initial hypothesis, there was no statistically significant correlation between the size and the year of diagnosis (r = - 0.084) (p-value = 0.168) (Figure 3).

## Discussion

In this study, we found that TC affects adults at a relatively young age, with a female predominance. The most common TC type was papillary, and male patients with papillary TC were older than female patients with no age difference in the other types. Most of the patients present at early TC stages. There was no trend toward smaller tumours in recent years in comparison to the past years.

Previous differentiated TC studies have shown that females were affected more often than males, which is consistent with our results [14-15]. The reason behind the higher incidence of TC in females is not well understood, but a possible theory is the effect of thyroid tumour cells’ exposure to endogenous estrogen hormone. As evident by experimental studies, estrogen’s action on thyroid cells is mediated through estrogen receptor alpha and beta. TC cells demonstrate the upregulation of alpha receptors, which stimulate tumorigenesis, and downregulation of beta receptors, which possibly act as a tumour suppressor.

Similar to the global data regarding thyroid cancer, the most common histological subtype was papillary, followed by follicular thyroid carcinoma [14-15]. The mean size in our study cohort was 2.3 cm, which is close to other studies reported from Saudi Arabia, where the mean size was 2.15 cm [4]. However, it is different from the trend around the world where smaller thyroid tumours are diagnosed. A 39-year-retrospective review from the SEER-9 database indicated that the majority of the newly diagnosed TCs were less than 2 cm in size [7]. In another study utilizing the Finnish Cancer Registry, the median tumour size was 2.5 cm in the decade between 1981 - 1991, and it dropped to 1.5 cm in the decade of 1992 - 2002 [16]. We are anticipating that the trend in Saudi Arabia will also exhibit a similar pattern in the upcoming few years, especially with the availability of more sophisticated medical investigations and as more Saudis gain access to private insurance. Of note, a recent Saudi study from Riyadh analysing TC trends concluded that the incidence of TC microcarcinomas has increased in the previous seven years in comparison to a steady overall TC incidence [17].

The mean age of diagnosis in our study was 40.6 years, which is comparable to the latest 2015 Saudi Cancer Registry, where the median age of TC was 39 years in females, and 44 in males and both are fairly younger than the reported age from data around the world [8]. In the US SEER-9 database, the mean age was 48 years, and in an epidemiological study from Korea, it was 46.9 years [7, 18]. The reason behind this is not yet fully explained. 

Up to our knowledge, there are no statistical data regarding poorly differentiated TC in Saudi Arabia. In our study, eight (2.81%) of TC cases were poorly differentiated. Six were female patients with a mean age of 45.3 years and two male patients with a mean age of 38.5 years. They were younger than the reported studies from Morocco and the US where the mean age at diagnosis of poorly differentiated TC was 60 and 57 years, respectively, with also female predominance [19-20]. Most of the TC patients in our study were diagnosed at Stage I. Similarly, Alzahrani et al. and Hussain et al. showed that more than half of cases diagnosed were in Stage I or at a localized stage [4, 6]. Improved screening quality by ultrasound and fine-needle aspiration is one of the factors thought to be contributing to the early detection rate [4, 9].

This study is among the first to utilize the 8th AJCC Staging System in Saudi Arabia to report TC data [13]. There are scarce data regarding poorly differentiated TC and NIFTP in our region. Therefore, with these data regarding NIFTP, differentiated, and poorly differentiated TC, we will assess in adding to the knowledge of TC epidemiology in Saudi Arabia.

The main limitation of this study was the retrospective nature with a relatively small number after excluding all the patients with incomplete histological data. In addition, it was a single institute experience.

## Conclusions

TC is one of the common malignancies in Saudi Arabia. Health care providers are encouraged to be mindful of its presentation and initial management. Nationwide data is required to provide better insight into this common disease.
